# Identification of a potential allosteric site of Golgi α-mannosidase II using computer-aided drug design

**DOI:** 10.1371/journal.pone.0216132

**Published:** 2019-05-08

**Authors:** Lina Irsheid, Thomas Wehler, Christoph Borek, Werner Kiefer, Ruth Brenk, Maria Elena Ortiz-Soto, Jürgen Seibel, Tanja Schirmeister

**Affiliations:** 1 Institute of Pharmacy and Biochemistry, University of Mainz, Mainz, Germany; 2 Department of Biomedicine, University of Bergen, Bergen, Norway; 3 Institute of Organic Chemistry, University of Würzburg, Würzburg, Germany; Weizmann Institute of Science, ISRAEL

## Abstract

Golgi α-mannosidase II (GMII) is a glycoside hydrolase playing a crucial role in the *N*-glycosylation pathway. In various tumour cell lines, the distribution of *N*-linked sugars on the cell surface is modified and correlates with the progression of tumour metastasis. GMII therefore is a possible molecular target for anticancer agents. Here, we describe the identification of a non-competitive GMII inhibitor using computer-aided drug design methods including identification of a possible allosteric binding site, pharmacophore search and virtual screening.

## Introduction

Selectins are carbohydrate-recognizing proteins and are essential for adhesion of metastasizing cancer cells [1]. Oncogenic cells often show an altered distribution of oligosaccharides on their cell surface. This modified distribution of carbohydrates is linked to disease progression, metastasis and poor clinical outcome. Targeting the glycosylation pathways is a possible approach to identify new anticancer agents [2]. Golgi α-mannosidase II (GMII) is one of these possible targets, since it is a key enzyme in *N*-glycan processing. Its inhibition has been linked to an induced tumour repression [3]. The alkaloid swainsonine, a well-known inhibitor of GMII, has exhibited anti-tumour effects in certain colon, breast, or skin cancer types [2]. However, due to the simultaneous inhibition of the structurally related lysosomal mannosidase, side effects restrict the clinical use of this potent inhibitor [4–6]. GMII belongs to the hydrolase family 38 [7] and catalyzes the cleavage of α-(1,6) and α-(1,3) glycosidic bonds between mannose residues in GlcNAcMan_5_GlcNAc_2_ (GlcNAc: *N*-acetyl-glucosamine; Man: mannose) resulting in the formation of GlcNAcMan_3_GlcNAc_2_ [5].

The catalytic mechanism of this enzyme is well understood and was subject of numerous studies [8–10]. The catalytic site of GMII essentially consists of two aspartate residues and a zinc cation. GMII acts as a retaining glycosidase and cleaves the glycosidic bonds in a two-step mechanism via a covalent glycosyl-enzyme transition state complex, thereby preserving the configuration of the anomeric C-atom [5,10–12].

Over the past two decades, only few novel ligands of glycosidases addressing the catalytic site have been identified. Known irreversible inhibitors are cyclophellitol (1991) [13] or 5-fluoro- (1996) [14] and 2-deoxy-2-fluoro-glycosides (1987) [15]. In a recent study from Overkleeft *et al*. [16], a new class of irreversible glycosidase inhibitors, cyclophellitol cyclosulfates, was introduced. However, since the discovery of the known reversible inhibitors of GMII, swainsonine (1982) [17] and mannostatin (1989) [18], no significant advances have been made with regard to this inhibitor class. This may be due to the unfavourable properties of this binding site, namely its small size and a high degree of negatively charged amino acids.

In this study, we aimed to identify new inhibitors by addressing a potential allosteric site. The GMII enzyme of *Drosophila melanogaster* (dGMII) with a high sequence identity to its human homologue (hGMII), was used as model enzyme [19]. Both dGMII and hGMII are 100% structurally identical in the radius of 6 Å around the central Zn^2+-^ion and share 41% sequence identity and 59% sequence similarity with regard to the entire protein according to our homology modeling studies. Furthermore, GMII has a high degree of sequence conservation among many eukaryotes [20]. Based on the structure, a potential binding site was identified and ligands were discovered using a combination of a protein-based pharmacophore model and molecular docking. A number of compounds were short-listed for enzymatic testing which resulted in one hit.

## Materials and methods

### Identification of a potential allosteric binding site of Golgi α-mannosidase II

The potential allosteric binding pocket of the GMII protein was identified by using the Site Finder application of MOE v2014.10 (Chemical Computing Group, Montreal, QC, Canada) [21], which uses a methodology based on so-called alpha spheres that are derived from the receptor atoms and describe the shape of the protein. Each alpha sphere was classified as either “hydrophilic” or “hydrophobic”. Collections of neighbouring spheres were clustered to produce a collection of sites, which were ranked according to the number of hydrophobic contacts with the receptor. In the rank-ordered list for each potential site the number of receptor atoms (size), the number of hydrophobic contacts, the number of side chain contacts and the PLB (Propensity for Ligand Binding) score [22] were reported along with a list of the residues containing the contacting atoms. During the site identification with the Site Finder application, the radii of a hypothetical hydrophilic hydrogen bonding atom (such as N or O) and of a hypothetical hydrophobic atom (such as C) were 1.4 Å and 1.8 Å, respectively. Each hydrophilic alpha sphere that has no neighbouring hydrophobic alpha sphere within 3 Å was discarded. Clusters of alpha spheres were merged, if at least two pairs of spheres were within 2.5 Å. Potential sites were only kept, if they consisted of at least three alpha spheres.

### Comparison of binding site properties–active site versus potential allosteric site

DogSite Scorer was used to compare the characteristics and the druggability of the active and the potential allosteric site. The DoGSite Scorer is a web server (http://dogsite.zbh.uni-hamburg.de) employed for identification and characterization of potential new binding pockets as well as the investigation of subpockets. General properties of the detected pockets such as the volume, the surface, the shape and the so-called druggability score are calculated. A SVM (support vector machine) model based on a discriminant analysis separates druggable from less druggable binding pockets. The druggability score is estimated for each pocket and subpocket resulting in values between 0 and 1, whereupon pockets with a score approaching 1 are assumed to be the more druggable ones [23,24].

### Receptor preparation and sphere set generation for virtual screening

The crystal structure of the *Drosophila melanogaster* GMII (dGMII) in complex with the inhibitor swainsonine (PDB code: 1HWW [3]) was chosen as the receptor for this study. Subsequently, polar hydrogen atoms were added to the protein and their positions were minimized using the AMBER force field [25] as implemented in MOE. Crystallographic water molecules and ions were removed in order to obtain a final receptor setup consisting of protein atoms only. Amber partial charges [26] were assigned to the protein atoms. Due to the lack of a reference ligand, the binding site was manually filled with dummy atoms. Spheres were placed into the proposed binding site around the dummy atoms to lower the effective dielectric constant in this region and to serve as matching points for ligand atoms during docking. The position of the sphere points in the final receptor setup used for docking was based on a simple cubic grid wrapping the dummy ligand atoms. The distance between neighbouring grid points was 2.2 Å at a maximum distance of 4 Å to any dummy atom. No sphere was closer than 2.5 Å to any receptor atom. Grids for storing the binding site information with respect to electrostatic and van der Waals potential as well as the solvent-excluded volume were calculated as described earlier [27,28].

### Small molecule preparation

For the compilation of a ligand database suitable for docking and the pharmacophore search a virtual in-house library of 5,574,182 commercially available small molecules was used. The molecules were stored in SMILES format [29] alongside with their calculated physicochemical properties (e.g. molecular weight, number of H-bond donors/acceptors, logP) [30]. The molecules were filtered and converted in a dockable format.

First, the library compounds were filtered for desired lead-like properties (Table 1).

**Table 1:** Compound selection criteria.

Selection criterion	Definition
SlogP	< 3.5 & > - 3.5
MW [g/mol]	< = 350 & > 250
TPSA [Å]	< 140
Rotatable bonds	< 7
Fused ring systems	< 4
Total charge	< 2 & > -2
Heavy atoms	< 30
Hydrogen-bond donors	< 6
Hydrogen-bond acceptors	< 11
Absence of unwanted functionalities	No unwanted groups^a^

^a^ mutagenic groups such as nitro groups, reactive groups such as thiols or Michael-acceptors, groups interfering with typical HTS assays such as quinones, groups with unfavourable pharmacokinetic properties such as sulfates [30].

Additionally, all compounds containing unwanted groups were removed. For the remaining molecules, likely protomers, tautomers and stereoisomers were calculated using in-house python scripts based on the OEToolkit (Openeye, Santa Fe, NM). Afterwards, three-dimensional structures and low energy conformations of all stereoisomers of compounds passing both the physicochemical property filter step and the pharmacophore search were generated with OMEGA [31] (version 2.4.6, Openeye, Santa Fe, NM). Partial charges and desolvation energies for the transfer of the small molecules from high dielectric medium (bulk solvent) to low dielectric medium within the binding site were calculated using AMSOL (http://comp.chem.umn.edu/amsol/) [32]. Finally, the multiple conformers of each molecule were aligned to their ring systems and stored in a hierarchical (flexibase) format [33].

### Pharmacophore search

The pharmacophore model was developed based on the spatial arrangement of the amino acids within the proposed allosteric binding pocket with special considerations to potential electrostatic or ionic interactions that could be formed. Both the pharmacophore model development and the pharmacophore search were carried out using MOE v.2014.10. In order to find suitable annotation points for the pharmacophore query, the standard “Unified” annotation scheme was applied. At the total, a set of 6 pharmacophore features were created. The hydrophobic feature is given a tolerance radius of 2.1 Å. In the region of E459, two features were represented: one expressed a hydrogen-bond donor or a cationic functionality within a radius of 2.4 Å and the counterpart of this feature, a hydrogen-bond acceptor, had a radius of 1.7 Å. The remaining three features all had radii of 1.0 Å. For the pharmacophore search, the stereoisomers of molecules passing the first filter step for desirable properties were converted into a three-dimensional format as described above (sd-file). Only those molecules with at least one low-energy conformation meeting the criteria of the pharmacophore search were kept for docking.

### Molecular docking

DOCK 3.6 was used to place the ligands into the potential allosteric binding site [28,32,33]. The following parameters were chosen for sampling of the ligand orientations: ligand and receptor bin sizes were 0.5 Å, whereas the overlap bin size was 0.4 Å and 0.5 Å for receptor and ligand bins, respectively. The distance tolerance for matching ligand atoms to the receptor matching sites were 1.2 Å. Each docking pose was scored for electrostatic and van der Waals complementarity and corrected for partial desolvation [33]. Only the best-scoring representation (correct protomer, tautomer and stereoisomer) for each docked molecule was stored in the output database.

### Docking analysis

The docking poses were stored in a MOE database alongside with their corresponding scores in kJ/mol. Subsequently, predicted binding modes were visualized for closer inspection to manually select the most promising candidate compounds. Ranking of the database entries was first performed according to the total docking score and in a second step according to the predicted ligand efficiency (LE) by using the following Eq (1):

$$LE=-1*\left(\frac{Score}{HAC}\right)$$ (1)

where “Score” represents the total docking score obtained and HAC is the heavy atom count.

The diagrams for the representation of predicted binding modes and the pharmacophore search were prepared using PyMOL (http://pymol.org) [34]. The figures comparing the properties of the active and the potential allosteric site with regard to charged and neutral regions within the binding sites were prepared by using the PyMOL script “resicolor.py” (https://pymolwiki.org/index.php/Resicolor). Hereby, acidic and basic residues were coloured red and blue, respectively. All other protein parts were coloured grey.

### Cloning and recombinant expression of the Golgi α-mannosidase II from *Drosophila melanogaster*

A *Drosophila* expression system (DES-Inducible/Secreted Kit) with a pCoHygro selection vector was purchased from Invitrogen (ThermoFisher, USA). CdCl_2_ and HIS-Select HF Nickel Affinity Gel both were supplied by Sigma-Aldrich (Germany). Affi-Gel Blue Gel was purchased from Bio-Rad (Germany) and the *Drosophila* media and buffers were purchased from Biowest and Sigma-Aldrich, respectively. A QuikChange Lightning Site-Directed Mutagenesis Kit from Agilent Genomics (Germany) was used for the introduction of base substitutions.

The Golgi α-mannosidase II gene was amplified by PCR from its corresponding cDNA of the *D*. *melanogaster* homologue while adding *NdeI/BglII* and *XhoI/EcoRI* restrictions sites at the 5´and 3´ends, respectively. Apart from the restriction sites introduced, the amplified gene already contained three internal *BglII* and *XhoI* recognition sequences. Thus, the gene was cloned into the vector pMAL-c5X flanked by *NdeI* and *EcoRI* restriction sites and the internal *BglII* and *XhoI* sequences were silenced by synonymous base substitution using the QuikChange Lightning Site-Directed Mutagenesis Kit. The gene with silenced internal restriction sites was cloned into the expression vector pMT/Bip/V5-HisA between *BglII* and *XhoI* restriction sites based on the DES expression system from Invitrogen (Inducible/secreted kit). Stable cell lines were selected via co-transfection of 3 mL *Drosophila* Schneider 2 (S2) cells (1.5 x 10^6^ cells/mL) with pMT/Bip/V5-HisA and pCoHygro as selection vectors in a 6 well plate. Cells were selected with 300–600 μg/mL hygromycin (final concentration) over 3 weeks at 28°C. Afterwards, cells were assayed for expression in 12 well plates employing different concentrations of CdCl_2_ (0–15 μM) over 24 and 48 h. The recombinant protein was detected in the growth medium by Western blot. 300 μg/mL Hygromycin was used in further experiments with suspension cultures and enzyme induction was performed with 15 μM CdCl_2_ over 48 h.

The Golgi α-mannosidase II was purified from the growth medium by immobilized metal affinity chromatography (IMAC) using the HIS-Select HF Nickel Affinity Gel after removal of fetal bovine serum (FBS) by incubation with the Affi-Gel Blue resin. The purified protein was aliquoted and stored at -80°C in 10 mM Tris-HCl (pH 8.3) supplemented with 100 mM NaCl (S1 Fig).

Following SDS-PAGE, proteins were transferred to a PVDF Immobilon-P Membrane (Merck, Germany). The expression level of the HIS-tagged Golgi *α*-mannosidase II was analyzed through binding and detection of monoclonal anti-HisG alkaline phosphatase conjugated antibodies (Life technologies, Germany).

### Enzyme assay

Inhibition assays with dGMII were performed at pH 5.75 in MES buffer (40 mM), supplemented with bovine serum albumin 1.1% (0.1% final concentration in MES buffer) and ZnSO_4_-solution 1.1% (0.1% final concentration in MES buffer) [35]. The recombinantly expressed and purified enzyme was added to a final concentration of 0.002 mg/mL per well. 4-Methylumbellifer-7-yl-α-D-mannopyranoside (4-MU-Man) [36] was synthesized [37] (S1 File and S2 Fig) and used as substrate with a final concentration of 1 mM per well. The total reaction volume was 50 μL and the mixture was incubated at 37°C for five minutes. The test compounds were dissolved in DMSO so that the DMSO concentration in the wells did not exceed 5% (v/v). The release of 4-methylumbelliferone was measured for 15 min by fluorescence spectroscopy using Greiner 96 Flat Bottom Black Polystyrol plates and a Tecan Reader Infinite F200 PRO equipped with a filter system (355 nm excitation and 485 nm emission). Assays in order to investigate the selectivity of compounds between different mannosidases were carried out on JBM. Assays concerning the selectivity between the desired mannosidase inhibition and a potential glucosidase inhibition were carried out on the β-glucosidase of sweet almonds using 4-methylumbelliferyl-β*-D*-glucopyranoside (4-MU-Glc) as substrate. The substrate 4-MU-Glc was synthesized [38] as described in the supplementary information (S1 File and S3 Fig). JBM and β-glucosidase from sweet almonds were purchased from Sigma Aldrich. The enzyme β-glucosidase from sweet almonds was diluted in citrate buffer in order to obtain a concentration of 0.04 mg/mL. The inhibition assays were carried out at a pH of 5.0 in citrate buffer (20 mM) [39]. The substrate concentration was chosen to be 1 mM per well in accordance with the results of several K_M_ value determinations since this concentration ensures good enzyme activity (K_M_ = 1.9 ± 0.1 mM). Inhibition assays JBM were performed in the same buffer as for dGMII. The final concentration of the enzyme per well was 0.003 mg/ml. The substrate 4-MU-Man was used with a final concentration of 0.4 mM per well. The conducted assays with JBM and the β-glucosidase from sweet almonds were performed as described for dGMII.

The reproducibility of the assay was assessed by determining the Z’-value as indicator for overall assay quality [40]. This was performed by measuring the residual reaction rate during 15 minutes with swainsonine (50 nM per well) and without swainsonine. The Z’-value was calculated to be 0.68 and therefore indicates good reproducibility since values between 0.5 and 1 are acceptable [40].

The most promising compounds according to the molecular modeling studies were tested at 300 μM final concentration except for compound **24** which was tested at 100 μM per well due to the small amount available. We considered the compounds as inactive when less than 40% of the enzyme activity was inhibited. The inhibition was calculated with the following Eq (2):

$$Inhibition\;(\%)=\left(1-\frac{activity\;(with\;inhibitor)}{activity\;(without\;inhibitor)}\right)∙100$$ (2)

K_M_ values were calculated using the Michaelis-Menten kinetics Eq (3)

$$v=\frac{v_{max}[S]}{K_{M}+[S]}$$ (3)

whereby v_max_ is the maximal velocity, [S] the substrate concentration and K_M_ the substrate concentration yielding a velocity of v_max_/2.

IC_50_ values were calculated using following Eq (4):

$$y=\frac{y_{max}-y_{min}}{1+{\left(\frac{\left[I\right]}{IC_{50}}\right)}^{S}}+y_{min}$$ (4)

whereby y_max_ is defined as 100% enzyme activity, y_min_ as 0% enzyme activity, *s* is defined as the slope of the linear part of the curves (Hill coefficient), and [I] represents the inhibitor concentration. For the K_M_ value determination, substrate concentrations ranging from 0.4 to 8.0 mM for dGMII and from 0.008 to 1.6 mM for JBM were used. For the IC_50_ determination of swainsonine, concentrations ranging from 6 to 120 nM were applied. For the IC_50_ determination of compound **18,** the substrate concentrations ranged from 10 to 1000 μM. In order to define the mode of inhibition, the determination of the IC_50_ values was carried out using different substrate concentrations ranging from 0.1 to 3 mM, since in case of non-competitive inhibition the IC_50_ value should be independent from the substrate concentration [41]. The program GraFit (5.0.13, Erithacus Software, Horley, Surrey, UK (2006)) was used to calculate the IC_50_ values.

## Results

### The potential allosteric Golgi α-mannosidase II binding site in comparison to the known active site

**Fig 1:**
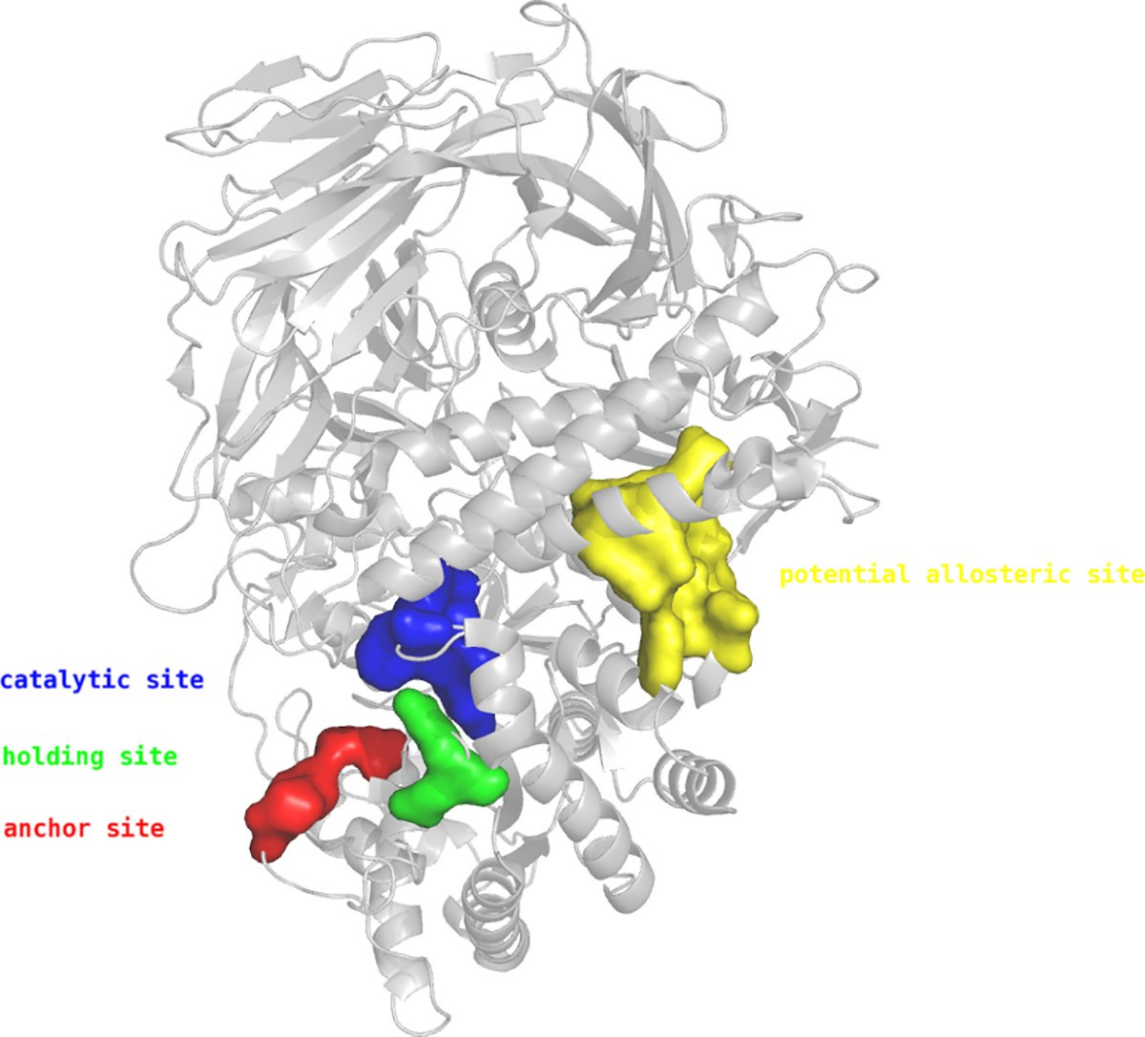
Overview of the different locations of the active site consisting of the catalytic site (blue), holding site (green), anchor site (red) with the potential allosteric site (yellow) within dGMII [3].

The protein structure of dGMII was analyzed to detect pockets that could be targeted by allosteric inhibitors. A total of 60 cavities were identified in the protein structure (PDB code: 1HWW [3]). After visual inspection of the most promising sites with regard to the size, the amino acid composition, the accessibility and the distance from the proposed site to the active site, one site has been chosen as potential allosteric site. This site consists of 102 receptor atoms, 29 are hydrophobic and 68 are side chain atoms. In contrast, the catalytic site is composed of 93 receptor atoms, 31 are hydrophobic and 75 are side chain atoms. Approximately 30 Å separate the catalytic site from the potential binding site when measured from the center of each site (Fig 1).

**Fig 2:**
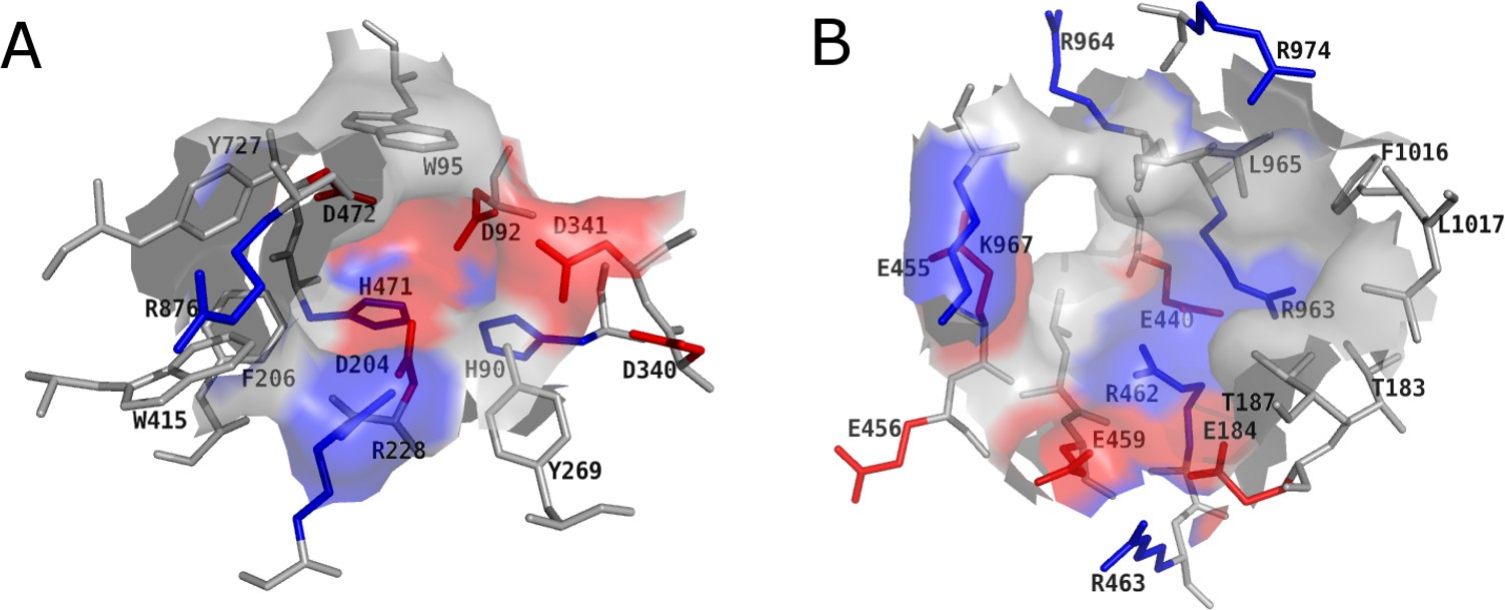
Colour-coded surface generated for the active site (A) and the identified pocket (B). Acidic and basic residues are coloured red and blue, respectively.

A comparison of the amino acid composition revealed some differences (Fig 2(A) and 2(B)).

The known catalytic site is compact, hydrophilic and predominantly negatively charged in the region where potential ligands are deeply buried (Fig 2(A)). The potential allosteric site seems to be more open compared to the active site. Furthermore, the amino acid composition is more balanced in terms of the distribution of negatively and positively charged residues (Fig 2(B)). The active site has a large negatively charged surface due to the acidic residues in its pocket (Fig 2(A)).

Amino acids such as D204, D340, D92 or D472 may accordingly interact with positively charged binding partners. The GMII is a metallo enzyme and contains a zinc ion in its active site. Noticeably, as with all zinc sites, the metal ion is enclosed by a shell of hydrophilic groups which is surrounded from a larger shell of hydrophobic groups [42]. On the other hand, the cavity of the potential allosteric site has a positively charged and a negatively charged surface patch. The positively charged patch is formed by the residues R462, R974 or K967 (Fig 2(B)). Amino acid residues such as E459, E184 or E440 are responsible for the negatively charged surface. In all other regions of the potential allosteric site neutral amino acids are predominant.

The druggability of the potential allosteric binding site identified was evaluated using the DoGSite Scorer considering the following properties: pocket volume, surface area, depth, druggability score and the apolar amino acid ratio (Table 2).

**Table 2:** Shape description and druggability prediction of the active and the potential allosteric site of GMII using DoGSiteScorer (PDB code: 1HWW [3]).

	Volume [Å^3^]	Surface [Å^2^]	Depth [Å]	Drugg-ability score	Apolar amino acid ratio
**Active site**	194.65	229.38	8.97	0.28	0.21
**Potential allosteric site**	879.26	820.14	22.46	0.83	0.46

With a druggability score of 0.83, the DoGSite Scorer predicted a high druggability for the potential allosteric site, since scores > 0.5 indicate a druggable binding site [23]. Furthermore, the properties of both sites differed significantly (Fig 2(A) and 2(B)). The larger pocket volume, higher depth, and a higher apolar amino acid ratio of the potential allosteric site are positive predictors of its druggability [23]. Thus, the potential allosteric site could potentially bind drug-like compounds.

### Pharmacophore hypothesis

**Fig 3:**
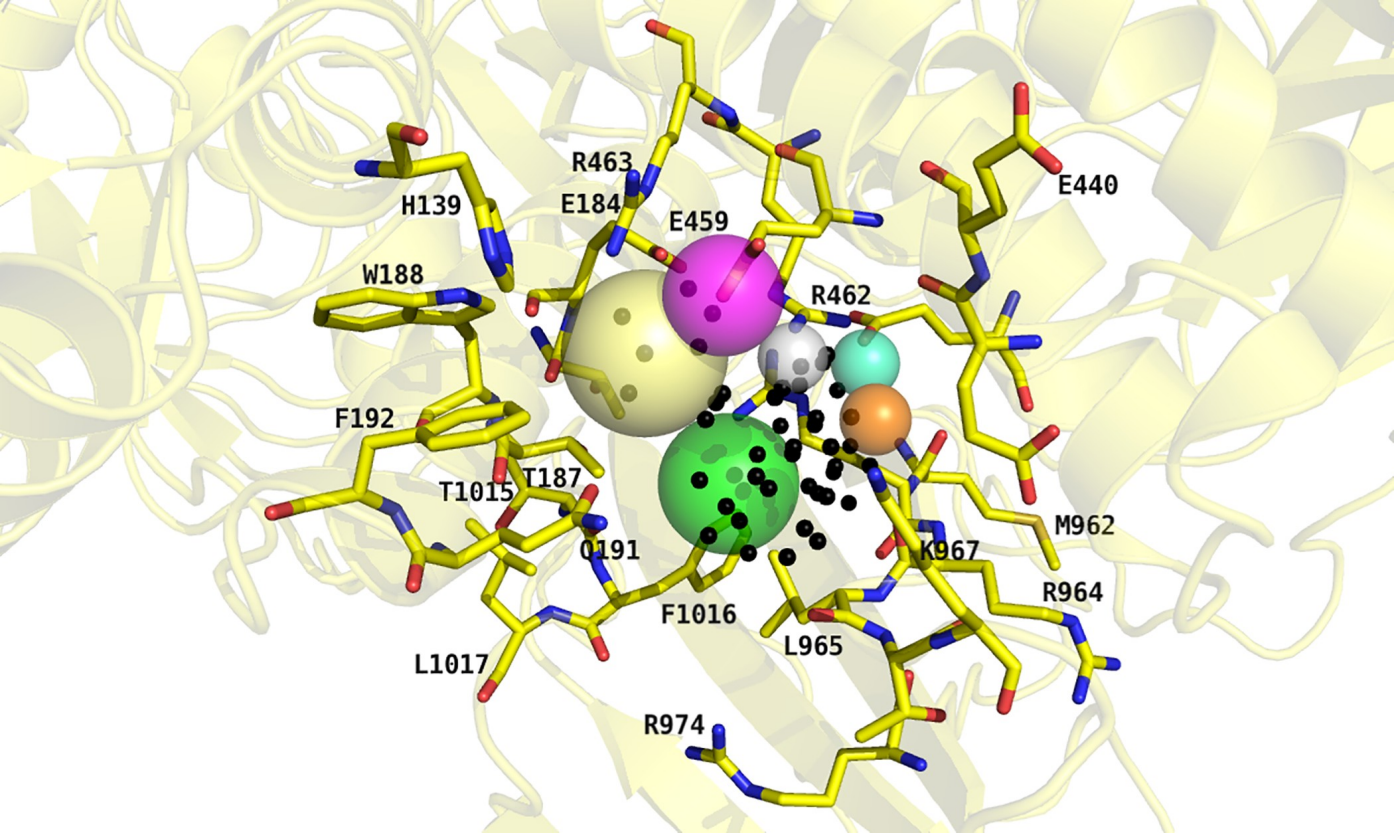
Pharmacophore hypothesis represented by colour-coded spheres. The volumes of the spheres define the positions and sizes of the corresponding electronic and steric features in matching ligand conformations. Binding site residues are displayed with carbon atoms in yellow colour. Dummy atoms used to define the binding site region are indicated by *black* spheres. The *pale-yellow* and *magenta* spheres, respectively, indicate the placement of a hydrogen-bond donor or cationic centre to interact with E459. The *green* sphere represents the area that had to be addressed with hydrophobic substituents. The desired position for either a hydrogen-bond donor or acceptor interacting with E459 and R462, respectively, is indicated by a *grey* sphere. The anionic centre with hydrogen bond acceptor properties is depicted by a *pale-cyan* sphere, whereas the *orange* sphere accounts for a hydrogen bond acceptor possibly interacting with K967.

In the absence of a known ligand for this potential allosteric binding pocket, the pharmacophore model was built based on its amino acid composition. Here, a special focus was set on charged amino acids buried inside the potential binding pocket to facilitate favourable electrostatic interactions between ligands and protein. The pharmacophore model included an excluded volume covering all receptor atoms to prevent a steric clash upon binding. Additionally, six other pharmacophore features were included in the model (Fig 3).

Two pharmacophore features were included to achieve interactions with E459. On the one hand, a hydrogen-bond donor or a cationic functionality was required close to the glutamate. On the other hand, the counterpart of this interaction, the carboxylic acid function of E459, was included as a hydrogen-bond acceptor. Further, a hydrophobic moiety was required close to the side chains of L965 and F1016. Another pharmacophore feature was located between E459 and R462 to either act as hydrogen-bond donor to E459 or as acceptor for R462. An additional feature determined an area of a hydrogen bond acceptor that may interact with the protonated side chain of K967 and the last feature represented the position of an anionic and hydrogen bond-accepting group presumably interacting with R462. In addition to the excluded volume area, the features accounting for the interaction of proposed ligands with E459 via either hydrogen bonding or electrostatic interactions and for the interaction network to E459 and/or R462, respectively, were determined as being mandatory. Ligand conformations could only pass the pharmacophore search if six out of seven features (with excluded volume) were matched.

Several million conformations of the > 600,000 molecules passing the first filter step for molecular properties were calculated. However, only the conformation with the lowest rmsd-value between the matching atom positions and the pharmacophore query points was kept in the results table. Altogether, 41,815 molecules passed this second filter step. Subsequently, these molecules were subjected to docking into the potential allosteric binding site.

### Virtual screening and docking results

For 36,607 compounds, a docking pose was obtained. Compounds for subsequent biological evaluation were selected according to the following criteria:

- total docking score (ΔG) predicted to be ≤ -25 kJ/mol
- predicted ligand efficiency (LE) as defined by Eq (1) ≥ 1 kJ/mol per heavy atom
- visual inspection of the predicted pose including an accurate analysis of the postulated protein-ligand interactions: hydrophobic parts should not be solvent-exposed and strong polar ligand groups were required to interact with at least one binding site residue via electrostatic and/or ion interactions in particularly with E459 und R462.

**Fig 4:**
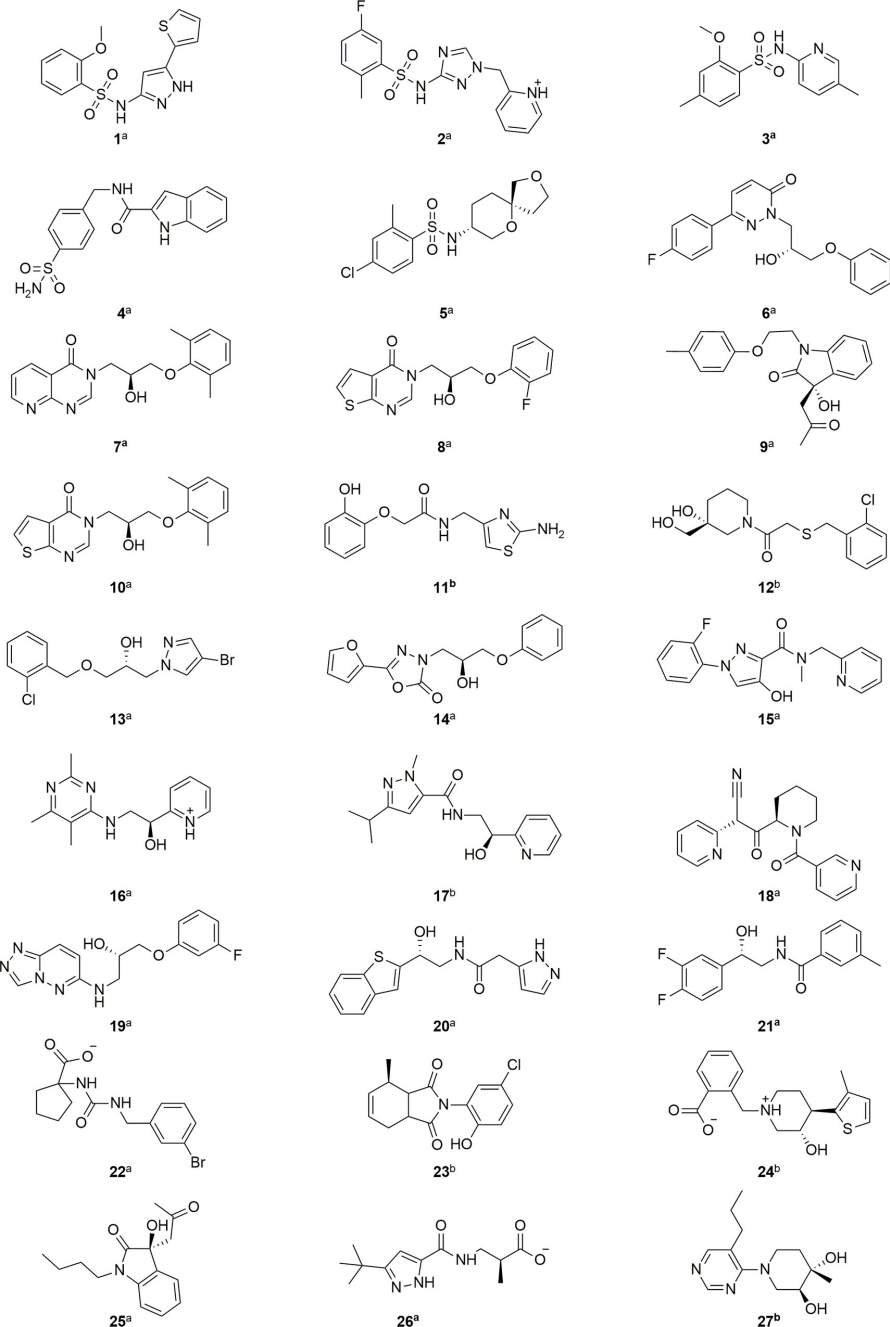
Molecular structures of virtual screening hits with corresponding compound number. ^a^ Compounds have been purchased from Enamine. ^b^ Compounds have been purchased from ChemBridge. The molecules are represented in the protonated form in which they were docked.

Based on the described criteria, compounds from different substance classes with scores from -69.11 to -25.66 kJ/mol and LEs reaching from 1.16 to 3.14 kJ×mol^-1^×HAC^-1^ were finally purchased. In total, 27 out of the 41,815 compounds were selected for testing (Fig 4).

These 27 compounds were either purchased from ChemBridge or Enamine and tested using a fluorometric enzyme assay. For the compounds with stereoisomerism, it was only possible to obtain racemic mixtures, although stereoisomers had been taken into account for the molecular docking.

### Results of enzyme assays, determination of K_M_ and IC_50_ values, and the mode of inhibition

**Fig 5:**
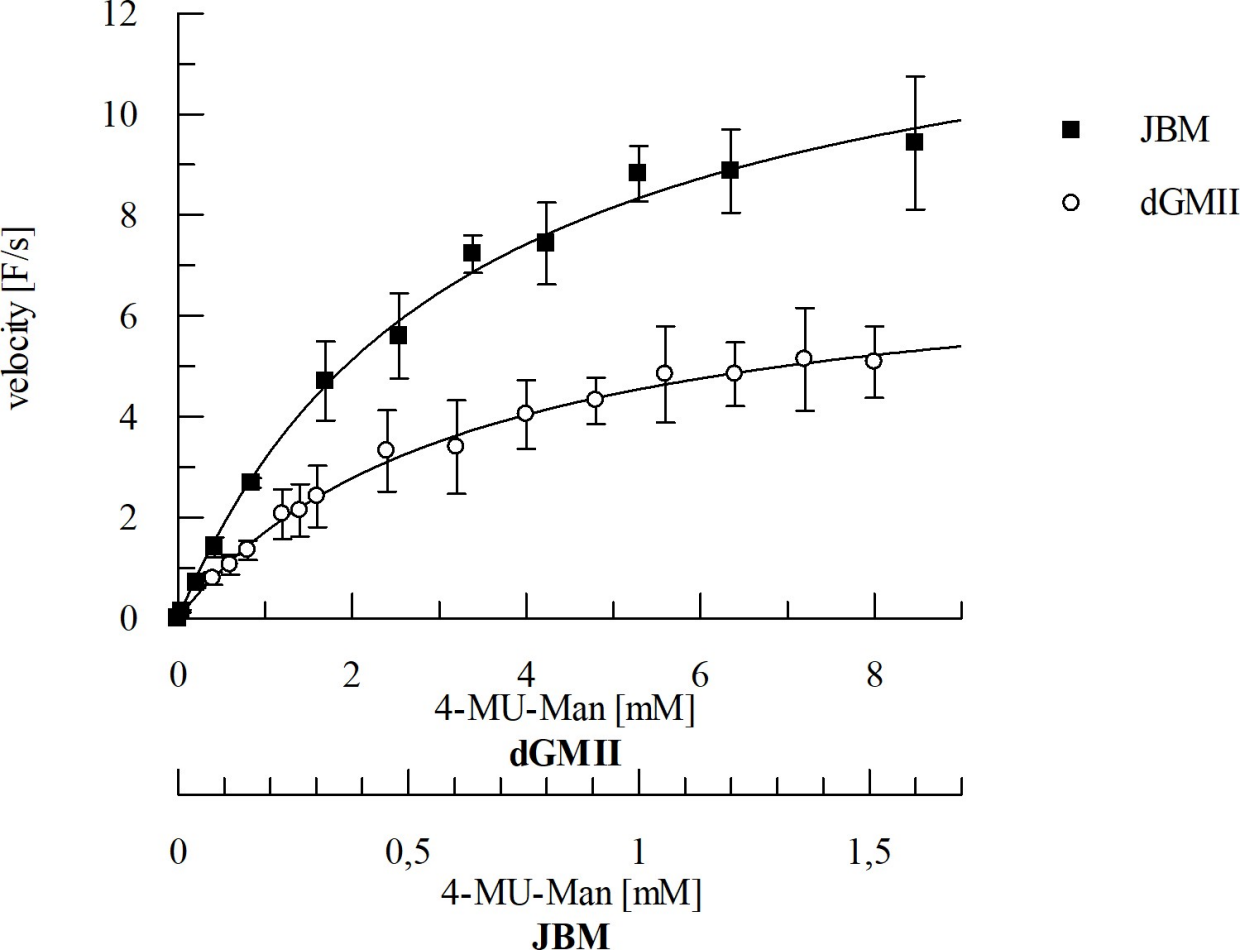
Example of the non-linear fit for the determination of the K_M_ values of the enzymes dGMII and JBM with 4-MU-Man as substrate. Non-linear regression was applied to the data using the Michaelis-Menten Eq (3).

The determined K_M_ values were 3.6 ± 0.3 mM and 0.6 ± 0.06 mM for dGMII and JBM, respectively (Fig 5).

**Fig 6:**
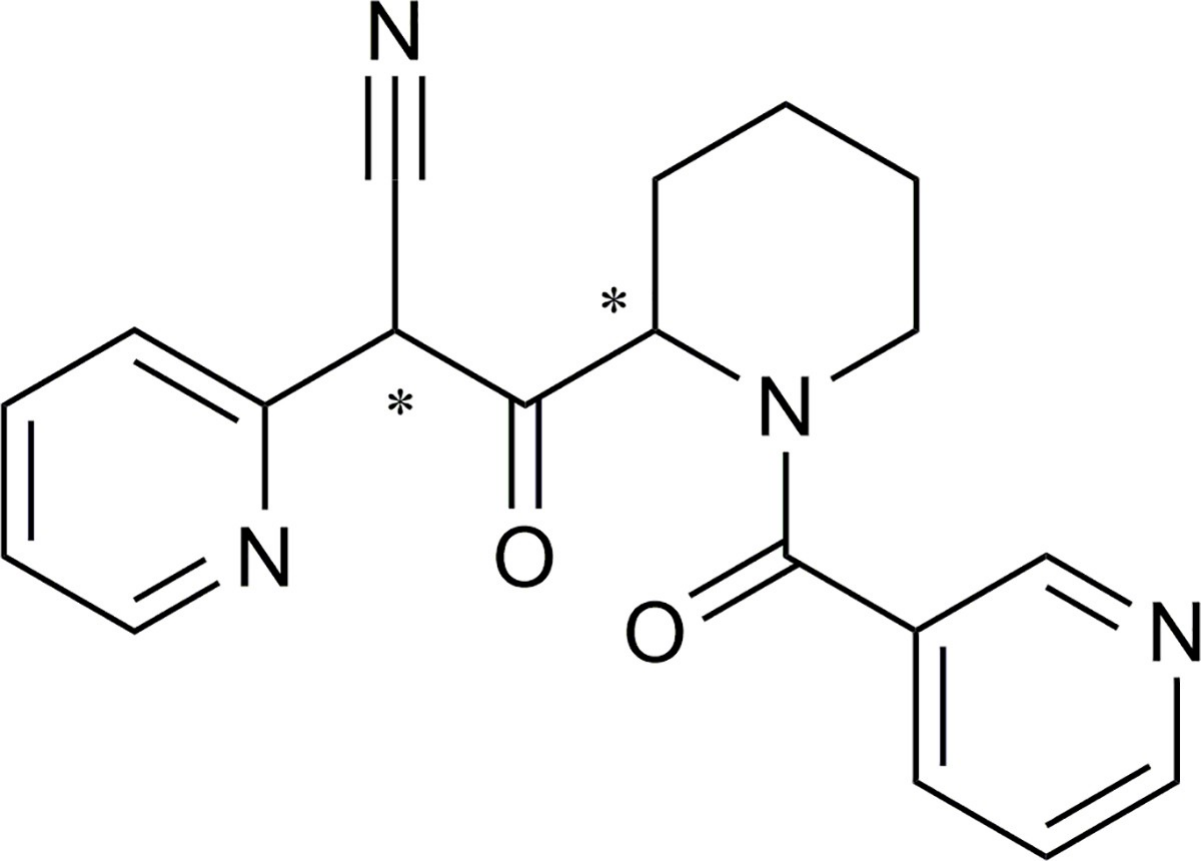
Chemical structure of compound 18.

Two out of the 27 compounds exhibited inhibition > 40% at 300 μM, namely the bis-pyridyl derivative **18 (**Fig 6**)**, (ca. 46% inhibition at 300 μM) and the benzothiophene **20** (ca. 41% inhibition at 300 μM).

**Fig 7:**
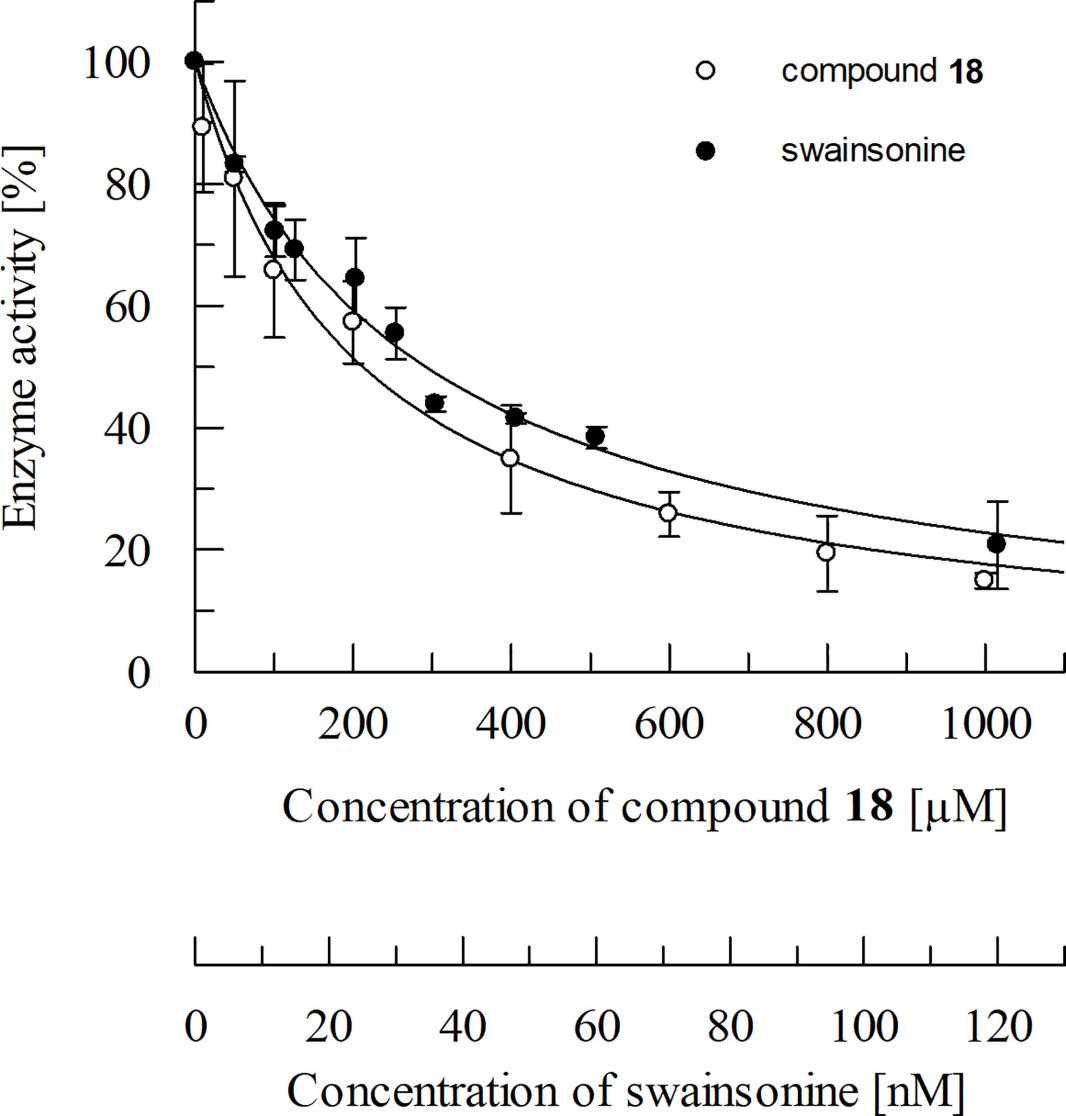
Example of an IC_50_ curve of compound 18 and of swainsonine at a substrate (4-MU-Man) concentration of 1.5 mM. Non-linear regression was applied to the data using Eq (4).

**Fig 8:**
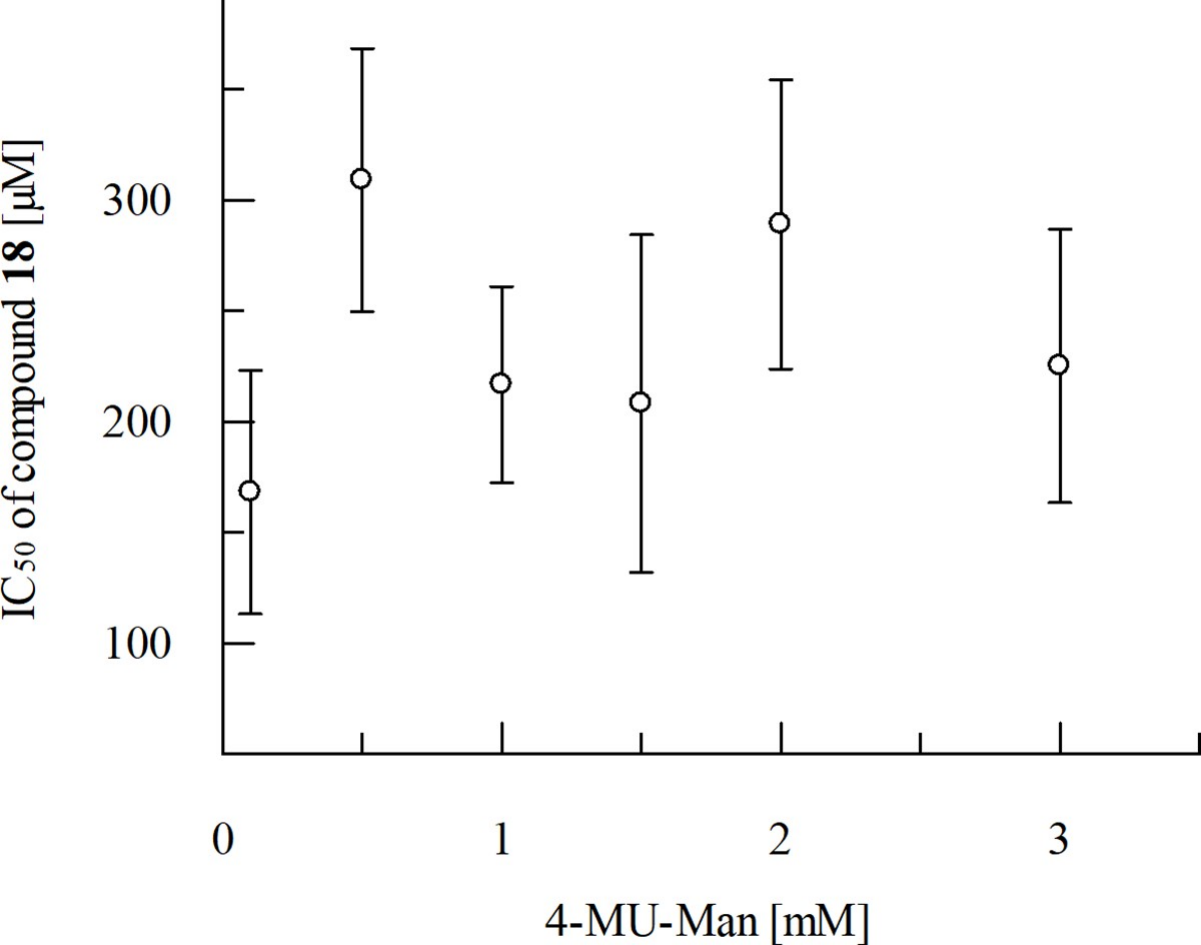
Effect of different substrate concentrations on the IC_50_ values of compound 18 from three independent measurements. The single sample t-Test [45] was applied to calculate the significance of the results. The *t*-value is 1.01. The value of p is 0.37. The result is not significant at p > 0.05. The p-value indicates that there is no statistically significant difference in the IC_50_ values of compound 18 at different substrate concentrations.

However, due to interference with the fluorometric assay compound **20** was not further pursued as the observed inhibition of GMII was expected to be caused by an experimental artefact. Compound **20** strongly interfered with the assay due to its auto-fluorescent properties. Independent of the concentration of compound **20**, large RFU values were detected. For compound **18,** the IC_50_ value was determined to be 216.7 ± 44.3 μM. The measured IC_50_ value of swainsonine was 34 ± 1.4 nM and is in the lower nanomolar range as described in the literature [43,44]. Assays with varying substrate concentrations showed the IC_50_ value of compound **18** to be independent of the substrate concentrations indicating non-competitive inhibition (Fig 7 and Fig 8).

**Fig 9:**
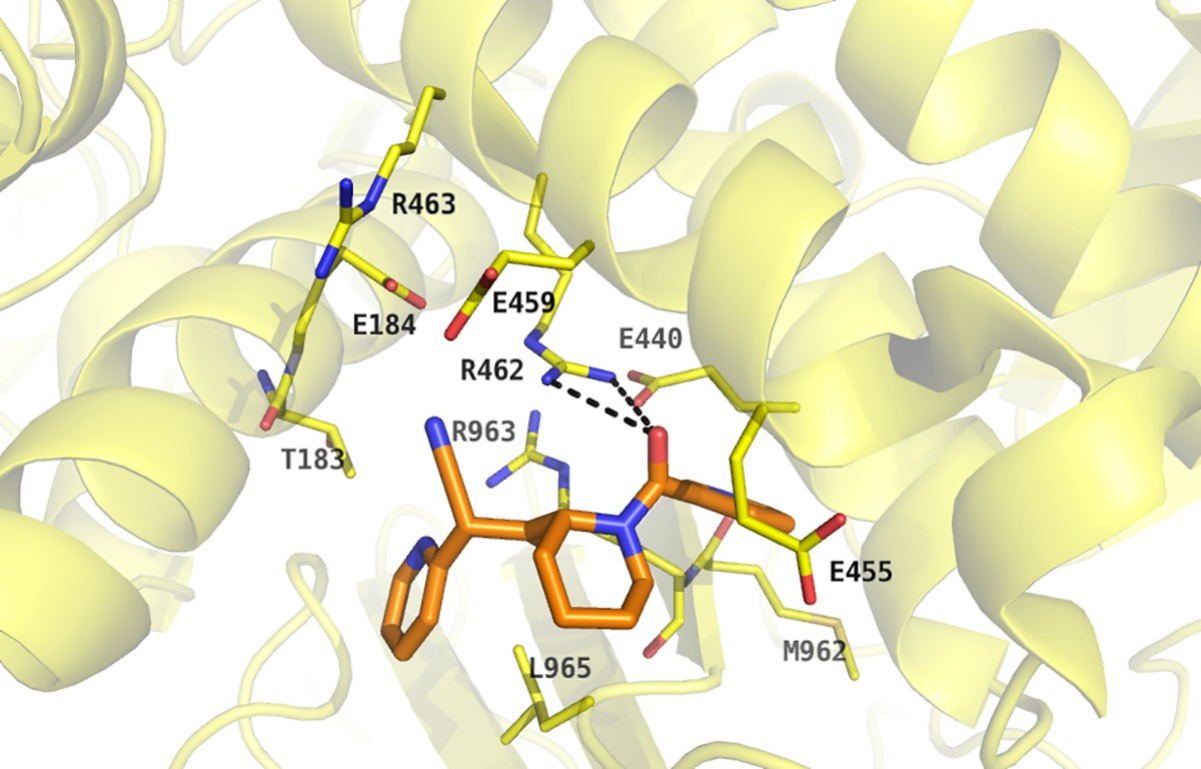
Predicted binding mode for compound 18. The GMII protein (PDB code: 1HWW [3]) is shown as *transparent*, *yellow-coloured cartoon* with binding site residues being displayed as stick representation with yellow-coloured carbon atoms. Compound 18 is represented with orange coloured carbon atoms and *black dashes* indicate potential hydrogen bonds between the virtual screening hit and the target protein.

In the predicted binding mode of compound 18 a bidentate hydrogen bond between the carbonyl oxygen of the amide bond and the protonated side chain of R462 is postulated (**Fig 9**).

**Fig 10:**
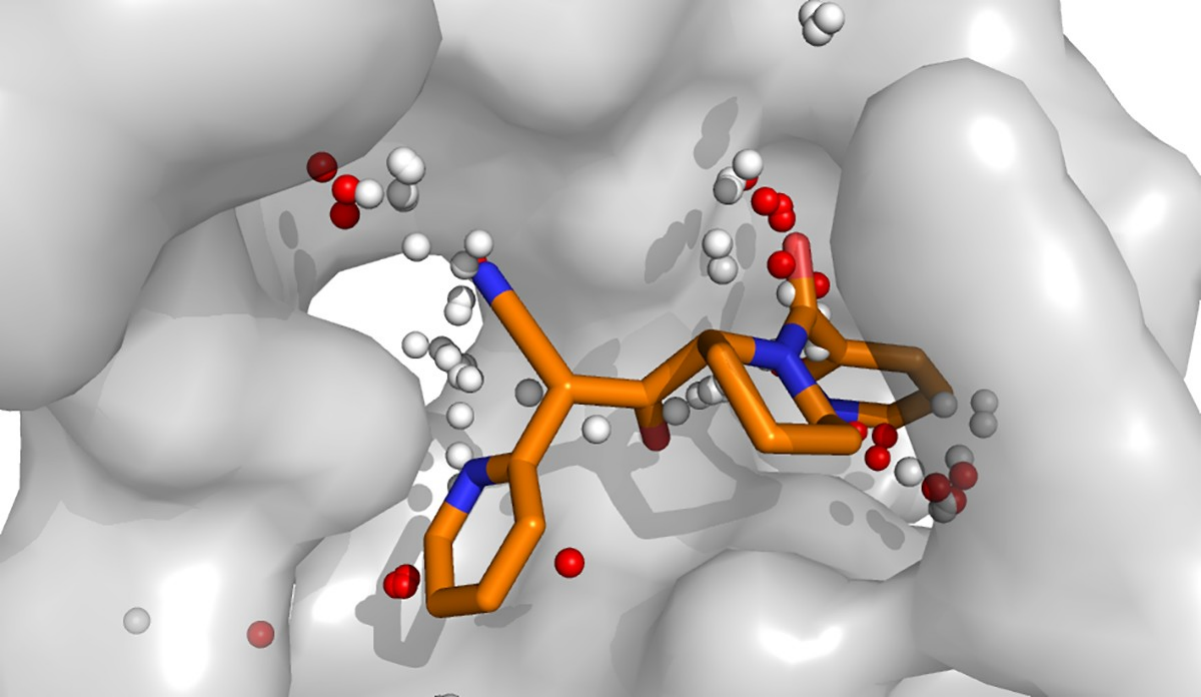
Comparison of the locations of alpha spheres and compound 18. Red alpha spheres indicate hydrophilic type of atoms and white hydrophobic groups favoured by the binding site regions.

The properties of the ligand atoms also match the properties of the dummy atoms created during binding site identification. Furthermore, the dummy atoms display areas that could be addressed during the optimization process (Fig 10).

In order to determine the selectivity of compound **18**, it was also tested for inhibition of the β-glucosidase of sweet almonds and JBM. At 300 μM no significant inhibition of the β-glucosidase of sweet almonds was observed. However, an inhibition of 70% of JBM activity was determined.

## Discussion

In this study, we described the identification of a potential allosteric site using the site finder application of MOE v2014.10. The DogSite Scorer predicted a higher druggability for this site in comparison to the known catalytic site, which is rather compact, hydrophilic and predominantly negatively charged in the region where potential ligands bind. Thus, its ligands are deeply buried. Thomas A. Halgren [46] has described the most suitable properties for the differentiation between ''druggable'', ''undruggable'' and ''difficult'' sites. The ''undruggable'' sites are usually much smaller and on average less protected from the solvent. Furthermore, these sites tend to display the most hydrophilic and the least hydrophobic character. According to these criteria, the catalytic site can be categorised as a ''difficult'' or ''undruggable'' site. Using DOCK 3.6, we performed a virtual screening aiming to identify new chemical scaffolds possibly interacting with this allosteric binding site. These studies revealed compound **18** as a first weak inhibitor of GMII (Fig 7). Since the IC_50_ value was independent of the substrate concentration (Fig 8) compound **18** is supposed to be a non-competitive inhibitor of GMII. This supports the hypothesis of having identified an allosteric site. According to the postulated binding mode, the polar interaction between the carbonyl oxygen of the amide bond and the protonated side chain of R462 might contribute to the ligand‘s binding affinity to its target (Fig 9). The commercially available β-glucosidase of sweet almonds was used as an off-target. Its primary structure consists of 542 amino acids compared to 1108 amino acids in the dGMII [47]. According to our homology modeling studies, both dGMII and the β-glucosidase share a low sequence identity of 12.2% and a similarity of 29.3%. Moreover, these enzymes belong to different glycoside hydrolase families with respect to retaining mechanism (GH38 and GH1, respectively) [7]. Compound **18** did not exhibit significant inhibition of the β-glucosidase of sweet almonds but showed inhibition activity against JBM (70% at 300 μM per well). The dGMII protein (PDB entry 1HWW [3]), homologous to hGMII, shares 23% identity and 40% similarity with JBM (PDB entry 6B9O [48]. The superposition of dGMII and JBM did not indicate the presence of a binding site similar to that identified in dGMII within the JBM structure. Similar to dGMII, the primary sequence of JBM suggests that this enzyme belongs to the glycoside hydrolase family 38 [49]. Thus, compound **18** can be considered as a weakly active inhibitor with some selectivity towards mannosidases in general. We identified compound **18** as a starting point for the identification of a novel class of GMII inhibitors which do not bind into the active site. Based on the inhibitory activity observed in the fluorometric assay, the ligand efficiency is approximately 0.20 kcal/mol. This is lower than the often applied threshold level of 0.30 kcal/mol for compounds suited for further optimization [50]. Despite the rather low efficiency of compound **18**, future investigations of the binding mode by e.g. site-directed mutagenesis studies might provide valuable insights into the properties that are required for ligands interacting with the potential allosteric binding site. This knowledge will then be useful to guide the design of analogues with improved affinities. Compound **18** could be structurally optimized in the future to address the amino acids R462, R463, K965, T187, E184 and E459 of the potential allosteric site of dGMII. For example, compound **18** could be optimized by addition of a substituent with cationic properties which enables the formation of strong ionic interactions with E184.

## Conclusion

The identified new GMII inhibitor compound **18 (**Fig 6**)** is a non-competitive inhibitor. This mode of inhibition underlines our proposed model of compounds binding to a potential allosteric pocket. Since the catalytic site has been studied in detail and it has been shown to be challenging to be addressed by molecular modeling [51], we opted for the identification of a potential allosteric site. The rational de novo design of inhibitors targeting this potential allosteric site is more challenging than the optimization of an already known inhibitor class or the screening for novel ligands binding to a site, for which ligand-bound three-dimensional structures are available. Thus, the identification of a first weak non-competitive inhibitor represents a first successful step into the development of novel GMII inhibitors.

## Supporting information

S1 Table List of score-ranked virtual screening hits. (PDF)

S1 File Synthesis of 4-methylumbellifer-7-yl-α-D-mannopyranoside (4-MU-Man) and Synthesis of 4-methylumbellifer-7-yl-β-D-glucopyranoside (4-MU-Glc). (DOCX)

S1 Fig SDS-PAGE of *Drosophila* proteins exported to the culture medium (A). Detection of the α-mannosidase II in the culture medium by western blot (B). Lines 1 to 5 show the expression of the α-mannosidase II in not transfected *Drosophila* cultures (1), Not induced, co-transfected cultures (2), transfected cultures with 5 (3), 10 (4) and 15 μM CdCl_2_ (5). α-mannosidase II was purified by incubating the culture medium with Affi-Gel Blue resin to eliminate the BSA from fetal bovine serum and subsequently by immobilized metal affinity chromatography (IMAC). Protein Molecular Weight Marker (Thermo Scientific Pierce, 14.4kDa to 116kDa) was used to identify the position of α-mannosidase II (121.54 kDa).
(TIF)

S2 Fig Synthesis of 4-methylumbellifer-7-yl-α-D-mannopyranoside (4-MU-Man). (TIF)

S3 Fig Synthesis of 4-methylumbellifer-7-yl-β-D-glucopyranoside (4-MU-Glc). (TIF)
