# Supplementary material for: Identification of a potential allosteric site of Golgi α-mannosidase II using computer-aided drug design
Source: PLoS One. 2019 May 8;14(5):e0216132. doi: 10.1371/journal.pone.0216132 (PMC6505943; doi:10.1371/journal.pone.0216132)
Supplement: S1 Table — (PDF) [file pone.0216132.s001.pdf]

Compound	Molecular structure	Score [kJ/mol]	Predicted LE* [kJ×mol^{-1}× HAC^{-1}]
**1**[a]	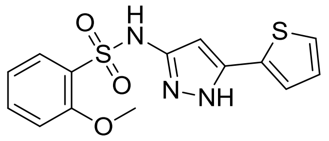	-69.11	3.14
**2**[a]	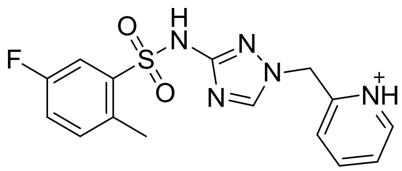	-60.48	2.52
**3**[a]	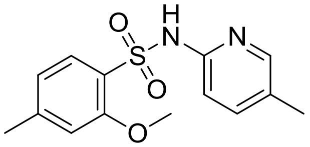	-56.03	2.80
**4**[a]	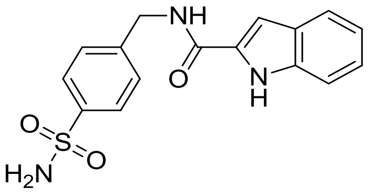	-53.66	2.33

Compound	Molecular structure	Score [kJ/mol]	Predicted LE* [kJ×mol^{-1}× HAC^{-1}]
**5**[a]	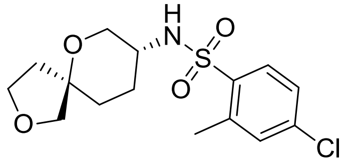	-46.75	2.12
**6**[a]	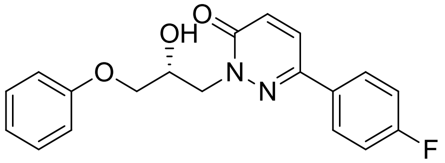	-39.96	1.60
**7**[a]	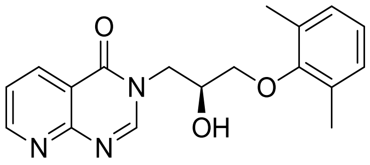	-38.81	1.62
**8**[a]	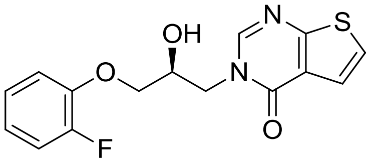	-37.87	1.72

Compound	Molecular structure	Score [kJ/mol]	Predicted LE* [kJ×mol^{-1}× HAC^{-1}]
**9**[a]	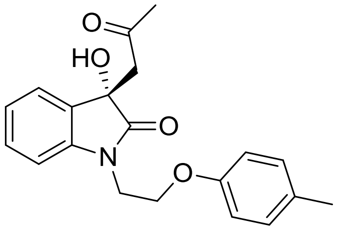	-37.63	1.50
**10**[a]	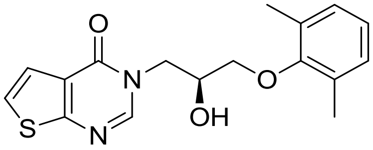	-37.27	1.62
**11**[b]	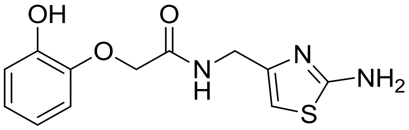	-35.78	1.88
**12**[b]	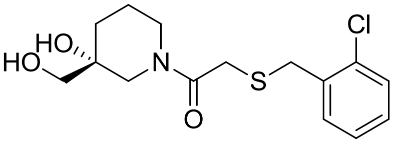	-35.76	1.70

Compound	Molecular structure	Score [kJ/mol]	Predicted LE* [kJ×mol^{-1}× HAC^{-1}]
**13**[a]	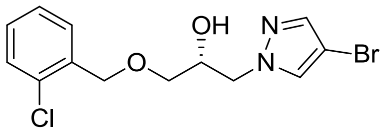	-35.29	1.86
**14**[a]	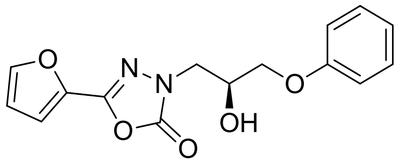	-34.53	1.57
**15**[a]	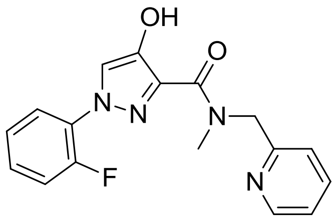	-32.38	1.35
**16**[b]	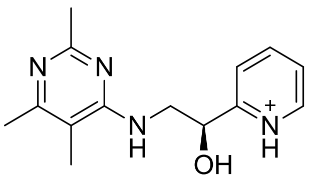	-32.32	1.70

Compound	Molecular structure	Score [kJ/mol]	Predicted LE* [kJ×mol^{-1}× HAC^{-1}]
**17**[b]	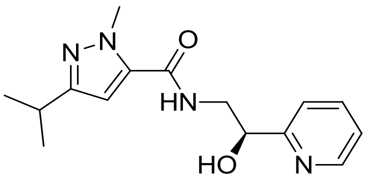	-31.43	1.50
**18**[a]	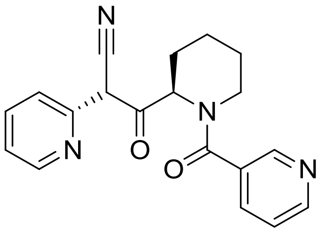	-31.38	1.25
**19**[a]	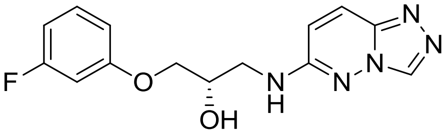	-31.37	1.43
**20**[a]	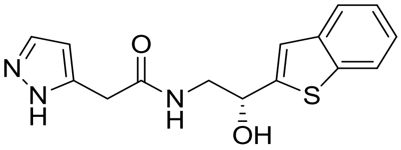	-31.25	1.49

Compound	Molecular structure	Score [kJ/mol]	Predicted LE* [kJ×mol^{-1}× HAC^{-1}]
**21**[a]	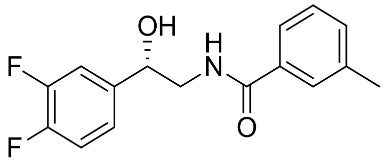	-31.24	1.49
**22**[a]	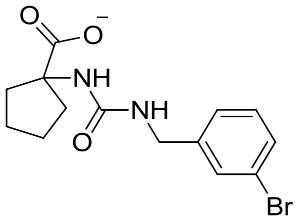	-31.22	1.56
**23**[b]	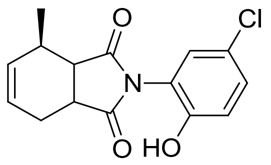	-31.08	1.55
**24**[b]	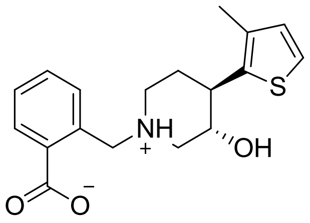	-26.77	1.16

Compound	Molecular structure	Score [kJ/mol]	Predicted LE* [kJ×mol^{-1}× HAC^{-1}]
**25**[a]	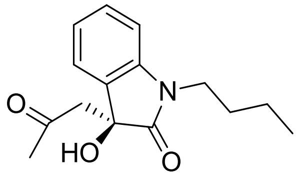	-26.40	1.40
**26**[a]	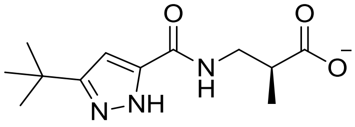	-25.95	1.44
**27**[b]	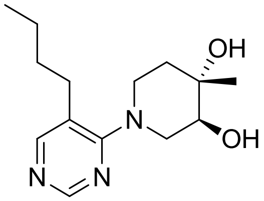	-25.66	1.50

* Predicted ligand efficiency (see equation (1))

** Inhibition (see equation (2)) at 300 µM (100 µM in case of compound **24**)

† Assay interference due to autofluorescence

[a] Compounds have been purchased from Enamine

[b] Compounds have been purchased from Chembridge
