# Supplementary material for: Identification of a potential allosteric site of Golgi α-mannosidase II using computer-aided drug design
Source: PLoS One. 2019 May 8;14(5):e0216132. doi: 10.1371/journal.pone.0216132 (PMC6505943; doi:10.1371/journal.pone.0216132)
Supplement: S1 File — (DOCX) [file pone.0216132.s002.docx]

**General information**: 4-Methylumbelliferone was purchased from Sigma-Aldrich. All other chemicals and solvents were from Fischer Scientific in standard grades and were used without further purification unless stated otherwise. TLC-plates were from Merck (TLC Silica gel 60 F254). All NMR-spectra were measured on a Bruker Fourier 300 spectrometer.

**Synthesis:** The synthesis of 4-MU-Man was first conducted according to the Koenigs-Knorr glycosylation procedure which uses acetylated glycosyl halides [1]. Due to very low yields of the coupling reaction (<10%) with peracetylated mannose bromide, an alternative route via the trichloreoacetimidate [2] was performed. This route starts with peracetylation of the monosaccharide in pure acetic anhydride, catalyzed by iodine. This method provides very pure products in short time and with quantitative yields at room temperature. The deacetylation of the anomeric oxygen atom with morpholine also delivered high purity product in high yields. The trichloracetimidate was obtained in 56 % yield. The coupling of 4-methylumbelliferone in dichloromethane with boron trifluoride diethyletherate delivered the desired product in high yield (88%). The final deprotection with sodium methanolate in dry methanol converted the precursor smoothly into the desired product. Thus, the here shown method yielded the substrate in 37% overall yield over five steps (S1 Fig).

**Synthesis of 1,2,3,4,6-penta-*O*-acetyl-*D*-mannopyranoside (1):** *D*-Mannose (10.0 g, 55,5 mmol) was dissolved in 50 ml of acetic anhydride. Under cooling in an ice bath 0.5 g iodine were added. After initial heat evolution the ice bath was removed after 10 min. The dark brown reaction mixture was stirred at room temperature for further 20 min. Then, aqueous sodium thiosulfate solution was added until the reaction mixture discoloured. Excess acetic anhydride was destroyed by cautious addition of solid sodium hydrogen carbonate. The mixture was extracted with dichloromethane, the organic layers were washed with sodium hydrogen carbonate solution, dried over magnesium sulfate, filtered off and distilled to yield (**5**) in quantitative yield as yellow viscous oil (mixture of *α*- and *β*-anomer with ratio of 25 % and 75 %) which was used in subsequent steps without further purification.

^1^H NMR of (**5**) (*β*-anomer) (300 MHz, chloroform-d) δ 6.08 (d, J = 1.8 Hz, 1H), 5.38 – 5.30 (m, 2H), 5.25 (t, J = 1.9 Hz, 1H), 4.30 – 4.23 (m, 1H), 4.13 (dd, J = 11.9, 2.3 Hz, 1H), 4.06 – 3.98 (m, 1H), 2.18 – 1.97 (m, 15H).

^1^H NMR of (**5**) (*α*-anomer) (300 MHz, chloroform-d) δ 5.85 (d, J = 1.1 Hz, 1H), 5.48 (dd, J = 3.2, 1.0 Hz, 1H), 5.31 – 5.27 (m, 1H), 5.12 (dd, J = 10.0, 3.3 Hz, 1H), 4.34 – 4.30 (m, 1H), 4.07 (s, 1H), 3.80 (ddd, J = 9.8, 5.3, 2.3 Hz, 1H), 2.22 – 1.98 (m, 15H).

^13^C NMR of (**5**) (*β*-anomer) (75 MHz, CDCl3) δ (5 x CO:170.65, 170.00, 169.75, 169.53, 168.07), 90.59, 70.60, 68.72, 68.32, 65.51, 62.09 (CH2), (5 x -CH3:20.87, 20.78, 20.73, 20.67, 20.65).

^13^C NMR of (**5**) *α*-anomer) (75 MHz, CDCl3) δ (5 x CO:170.66, 170.21, 169.80, 169.58, 168.38), 90.41, 77.24, 73.29, 70.64, 68.17, 65.37 (CH2), (5 x -CH3:20.76, 20.73, 20.68, 20.65, 20.55).

**Synthesis of 2,3,4,6-tetra-*O*-acetyl-*D*-mannopyranoside (2):** 9.8 g (26.1 mmol) of (**5**) were dissolved in 160 ml dry dichloromethane. After addition of 4 equivalents of morpholine (8.7 g, 100 mmol) the reaction mixture was stirred under argon atmosphere overnight. Then, the mixture was extracted two times with 1 M aqueous hydrochloric acid and three times with water. The organic layer was dried over MgSO4, filtered off and the solvent was distilled under reduced pressure to yield 8,0 g (23 mmol, 92 %) of the product as yellowish viscous oil. This product was used for subsequent reactions without further purification.

^1^H NMR (300 MHz, Chloroform-d) δ 5.42 (dd, J = 10.0, 3.2 Hz, 1H), 5.35 – 5.21 (m, 4H), 4.32 – 4.17 (m, 2H), 4.20 – 4.09 (m, 1H), 2.16 (s, 3H), 2.11 (s, 3H), 2.05 (s, 3H), 2.00 (s, 3H).

^13^C NMR (75 MHz, CDCl3) δ 170.80, 170.17, 169.99, 169.79, 92.24, 69.93, 68.70, 68.56, 66.14, 62.55, 20.93, 20.80, 20.74, 20.72.

**Synthesis of 2,3,4,6-tera-*O*-acetyl-*D*-mannopyranosyltrichloroacetimidate (3)**: 13.6 g (39 mmol) of (**6**), were dissolved in 150 ml dry dichloromethane. At 0 °C, 14 g (10 ml, 100 mmol, 2.5 equivalents trichloroacetimidate were added in small portions. The mixture was stirred for 1 h at this temperature and overnight at room temperature. Then, a small excess of diazabicyclo[5.4.0]undec-7-ene (DBU) (6.3 g, 6.4 ml, 41 mmol, 1.05 equivalents) were added and the reaction was stirred further 30 min. The mixture was then concentrated under reduced pressure and purified by column chromatography (silica gel, eluent ethylacetate:cyclohexane 2:1) to yield 10.7 g (21.8 mmol, 56 %) of the desired product as off white solid. Rf = 0.59 (ethylacetate:cylohexane 2:1), stained with anisaldehyde / sulfuric acid

^1^H NMR (300 MHz, Chloroform-d) δ 8.79 (s, 1H), 6.27 (s, 1H), 5.50 – 5.32 (m, 3H), 4.33 – 4.10 (m, 3H), 2.20 (s, 3H), 2.08 (s, 3H), 2.06 (s, 3H), 2.01 (s, 3H).

^13^C NMR (75 MHz, CDCl3) δ 170.72, 169.95, 169.88, 169.76, 159.87, 94.62, 71.31, 68.91, 67.98, 65.48, 62.16, 20.93, 20.84, 20.76.

**Synthesis of 4-methylumbellifer-7-yl-2,3,4,6-tetra-*O*-acetyl-*α-D*-mannopyranoside (4):** 10.7 g (21.8 mmol) of (**7**) were dissolved in 50 ml dry dichloromethane and, after addition of 7.4 g (42 mmol, 2 equivalents) 7-hydroxy-4-methyl umbelliferone and 670 mg (0.6 ml, 4.7 mmol, 0.2 äq) boron trifluoride diethyletherate the reaction mixture was stirred at room temperature for 3 h, after which most of the starting materials had reacted (TLC control). Excess of 7-hydroxy-4-methyl umbelliferone was filtered off, the filtrate was washed with water, dried over MgSO_2_ and concentrated under reduced pressure. The residue was purified by column chromatography (silica gel, gradient ethylacetate:cyclohexan 3:1 🡪 1:1 🡪 pure ethylacetate to yield 8.8 g of the product as off white solid (17.4 mmol, 79.8 %) beside 1 g (2 mmol, 9 %) of slightly impure product as yellowish solid which was used for the following step without further purification. Rf = 0.45 (ethylacetate:cylohexane 1:1, UV-active + stained with anisaldehyde / sulfuric acid).

^1^H NMR (300 MHz, Chloroform-d) δ 7.54 (d, J = 8.8 Hz, 1H), 7.12 (d, J = 2.0 Hz, 1H), 7.03 (dd, J = 8.8, 2.1 Hz, 1H), 6.20 (s, 1H), 5.58 (s, 1H), 5.57 – 5.52 (m, 1H), 5.47 (s, 1H), 5.38 (t, J = 10.0 Hz, 1H), 4.29 (dd, J = 12.4, 5.8 Hz, 1H), 4.10 – 4.00 (m, 2H), 2.42 (s, 3H), 2.22 (s, 3H), 2.05 (s, 9H).

**Synthesis of 4-methylumbellifer-7-yl-*α-D*-mannopyranoside (5):** 8.8 g (17.4 mmol) of (**8**) and 1.8 g (33 mmol) sodium methanolate were stirred in 50 ml of dry methanol at room temperature for 1 h. The reaction mixture was neutralized with Amberlite© (H+-form) and filtered off. The crude product was purified by column chromatography (silica gel, isopropanol:chloroform 1:1) to yield 4.5 g (13.3 mmol, 76.4 %) of the product as white solid.

^1^H NMR (300 MHz, DMSO-d6) δ 7.65 (d, J = 8.6 Hz, 1H), 7.14 – 6.97 (m, 2H), 6.20 (s, 1H), 5.53 (s, 1H), 3.87 (s, 1H), 3.70 (dd, J = 9.1, 3.0 Hz, 1H), 3.60 (d, J = 11.2 Hz, 1H), 3.58 – 3.46 (m, 1H), 3.46 (dd, J = 10.6, 4.7 Hz, 1H), 3.40 – 3.30 (m, 1H), 2.36 (s, 3H).

^13^C NMR (75 MHz, DMSO) δ 160.13, 159.14, 154.36, 153.31, 126.44, 114.15, 113.71, 111.76, 103.66, 98.83, 75.39, 70.64, 69.87, 66.66, 61.04, 18.18.

**Synthesis:** The synthesis of 4-MU-Glc (**4**) was carried out via Koenigs-Knorr glycosylation, starting with bromination of peracetylated glucose yielding 65% of the *α*-configured product [1]. The coupling of 4-methylumbelliferone requires freshly prepared silver carbonate and purification by column chromatography to give the glycoside (**3**) in 90 % yield. Deprotection was carried out with an access of sodium methanolate and yielded 56 % of the substrate (**4**) in high purity (S2 Fig).

**Synthesis of 1-bromo-2,3,4,6-tetra-*O*-acetyl-*D*-glucopyranoside (2):** 8 g (20,5 mmol) of peracetylated *D*-glucose (**1**) were dissolved in 40 mL of dry dichloromethane under argon atmosphere and mixed with 16.2 ml 33% hydrobromic acid in acetic acid and stirred at room temperature over night. The mixture was poured on ice, the organic phase was separated, the aqueous layer was extracted two times with dichloromethane. The combined organic layers were washed with water (3x), concentrated hydrogencarbonate solution (2x) and finally with brine. After drying over magnesium sulfate the mixture was concentrated under reduced pressure to yield a yellow oil. The product was recrystallized from a mixture of diethyl ether, diisopropyl ether and petrol ether in the freezer to yield 5.5 g product as white crystals (13,4 mmol, 65,4%) exclusively in α-form. Rf = 0.69 (petrol ether:ethyl acetate 1:1), stained with anisaldehyde / sulfuric acid.

^1^H NMR of (1) (300 MHz, Chloroform-d) δ 6.61 (d, J = 4.0 Hz, 1H), 5.56 (t, J = 9.7 Hz, 1H), 5.16 (t, J = 9.8 Hz, 1H), 4.84 (dd, J = 10.0, 4.0 Hz, 1H), 4.41 – 4.23 (m, 2H), 4.13 (d, J = 10.5 Hz, 1H), 2.10 (s, 6H), 2.05 (s, 3H), 2.04 (s, 3H).

**Synthesis of 4-methylumbellifer-7-yl-2,3,4,6-tetra-*O*-acetyl-*β-D*-glucopyranoside (3):** 2.4 g (5.77 mmol, 3.4 eq) of (**2**), were dissolved in 20 ml of dry dichloromethane. A mixture of 300 mg 4-methylumbelliferone (1.70 mmol, 1 eq), 800 mg of freshly prepared silver carbonate and one drop of pyridine in 20 ml acetonitrile were added. The suspension was stirred under argon atmosphere and protected from light for two days. Then the mixture was evaporated under reduced pressure to form a yellow foam which was dissolved in dichloromethane and subjected to column chromatography (cyclohexane:ethyl acetate 1:1) to yield a yellow oil which still contained considerable amounts of unreacted 4-methylumbelliferone. It was dissolved in a small amount of diethyl ether and pentane was added. After cooling to –80 °C 4-methylumbelliferone started to crystallize. After two days, the remaining solution was nearly colourless and was separated from the crystals. After evaporation of the solvent a yellowish foam (790 mg, 1.16 mmol, 91%) remained, containing the product in sufficient purity for the subsequent deprotection. Rf = 0.40 (petrol ether:ethyl acetate 1:1), stained with anisaldehyde / sulfuric acid

^1^H NMR (300 MHz, Chloroform-d) δ 7.51 (d, J = 8.6 Hz, 1H), 7.00 – 6.86 (m, 2H), 6.19 (s, 1H), 5.31 (dd, J = 6.3, 2.9 Hz, 2H), 5.20 – 5.12 (m, 2H), 4.35 – 4.06 (m, 3H), 3.96 – 3.87 (m, 1H), 2.40 (s, 3H), 2.11 (s, 3H), 2.06 (s, 6H), 2.03 (s, 3H).

^13^C NMR (75 MHz, CDCl_3_) δ 170.64, 170.19, 169.42, 169.27, 160.81, 159.19, 154.83, 152.22, 125.74, 115.53, 114.03, 113.20, 103.95, 98.35, 72.57, 72.42, 70.94, 68.07, 61.82, 20.71, 20.62, 20.60, 18.70.

**Synthesis of 4-methylumbellifer-7-yl-*β-D*-glucopyranoside (4):** 780 mg (1,54 mmol) of (**3**) were dissolved in 40 mg of absolute methanol and stirred with 300 mg of sodium methanolate overnight. The reaction mixture was neutralized with Amberlite© (H+-form) and filtered off. The crude product was purified by column chromatography (silica gel, isopropanol:chloroform 1:1) to yield 293 mg (0,87 mmol, 56,6 %) of the product as white solid.

^1^H NMR (300 MHz, DMSO-d6) δ 7.58 (d, J = 8.8 Hz, 1H), 6.98 (dd, J = 8.8, 2.0 Hz, 1H), 6.91 (d, J = 2.0 Hz, 1H), 6.10 (s, 1H), 4.98 (d, J = 7.0 Hz, 1H), 3.73 (d, J = 11.3 Hz, 1H), 3.50 (ddd, J = 19.5, 13.4, 5.2 Hz, 2H), 3.38 (t, J = 6.6 Hz, 2H), 3.25 (t, J = 8.8 Hz, 1H), 2.28 (s, 3H).

^13^C NMR (75 MHz, DMSO) δ 163.93, 161.08, 156.54, 155.23, 128.00, 115.98, 115.21, 112.65, 104.64, 100.94, 77.63, 76.85, 74.09, 70.67, 61.77, 19.41.

**References**

1. Malet C, Viladot JL, Ochoa A, Gallégo B, Brosa C, Planas A. Synthesis of 4-methylumbelliferyl-β-d-glucan oligosaccharides as specific chromophoric substrates of (1 → 3),(1 → 4)-β-d-glucan 4-glucanohydrolases. Carbohydr Res. 1995;274(C):285–301.

2. Touisni N, Maresca A, McDonald PC, Lou Y, Scozzafava A, Dedhar S, et al. Glycosyl coumarin carbonic anhydrase IX and XII inhibitors strongly attenuate the growth of primary breast tumors. J Med Chem. 2011;54(24):8271–7.
