# Supplementary figures and images for: Identification of a potential allosteric site of Golgi α-mannosidase II using computer-aided drug design
Source: PLoS One. 2019 May 8;14(5):e0216132. doi: 10.1371/journal.pone.0216132 (PMC6505943; doi:10.1371/journal.pone.0216132)

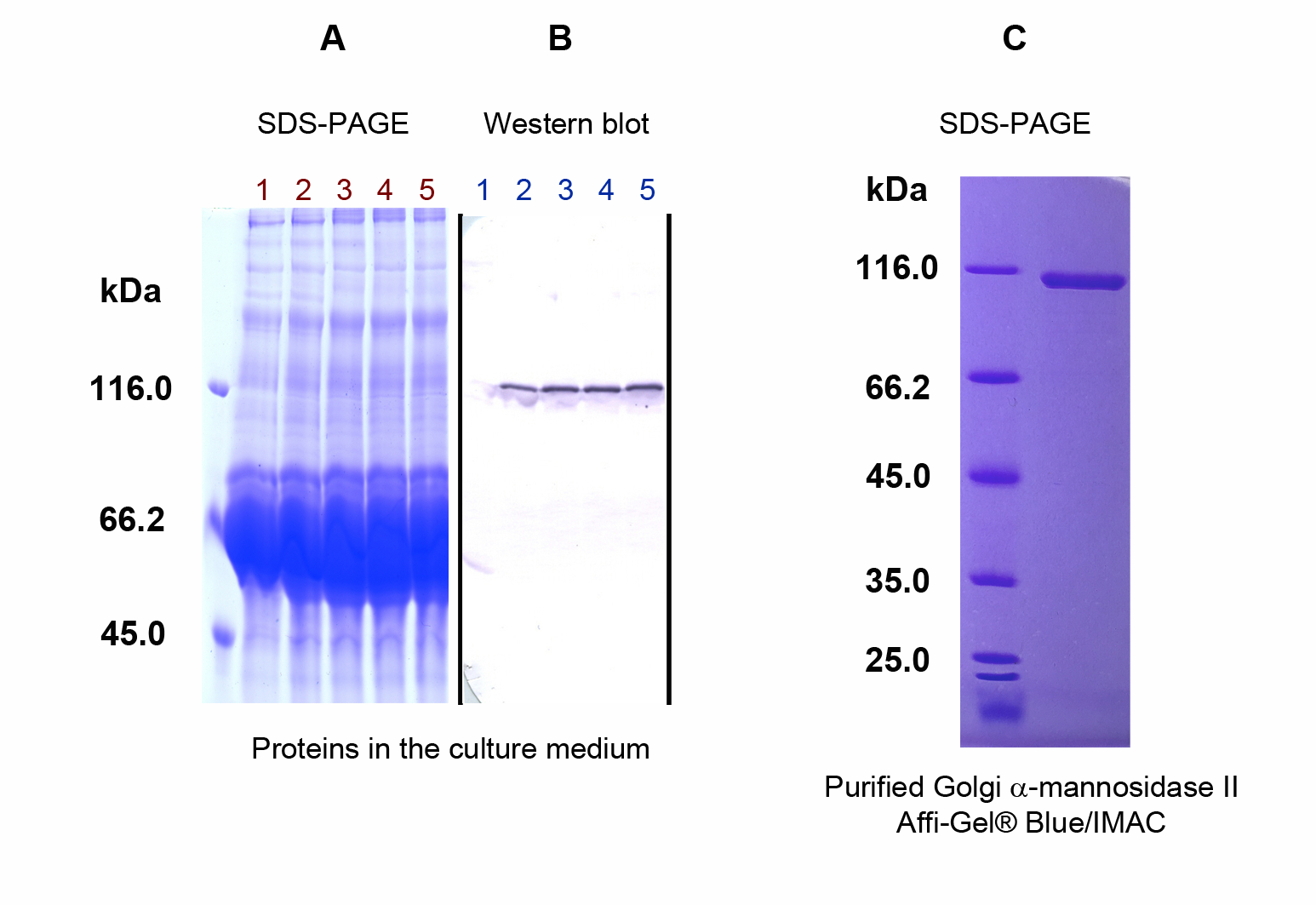

Supplement: S1 Fig — SDS-PAGE of Drosophila proteins exported to the culture medium (A). Detection of the α-mannosidase II in the culture medium by western blot (B). Lines 1 to 5 show the expression of the α-mannosidase II in not transfected Drosophila cultures (1), Not induced, co-transfected cultures (2), transfected cultures with 5 (3), 10 (4) and 15 μM CdCl2 (5). α-mannosidase II was purified by incubating the culture medium with Affi-Gel Blue resin to eliminate the BSA from fetal bovine serum and subsequently by immobilized metal affinity chromatography (IMAC). Protein Molecular Weight Marker (Thermo Scientific Pierce, 14.4kDa to 116kDa) was used to identify the position of α-mannosidase II (121.54 kDa). (TIF) [file pone.0216132.s003.tif]

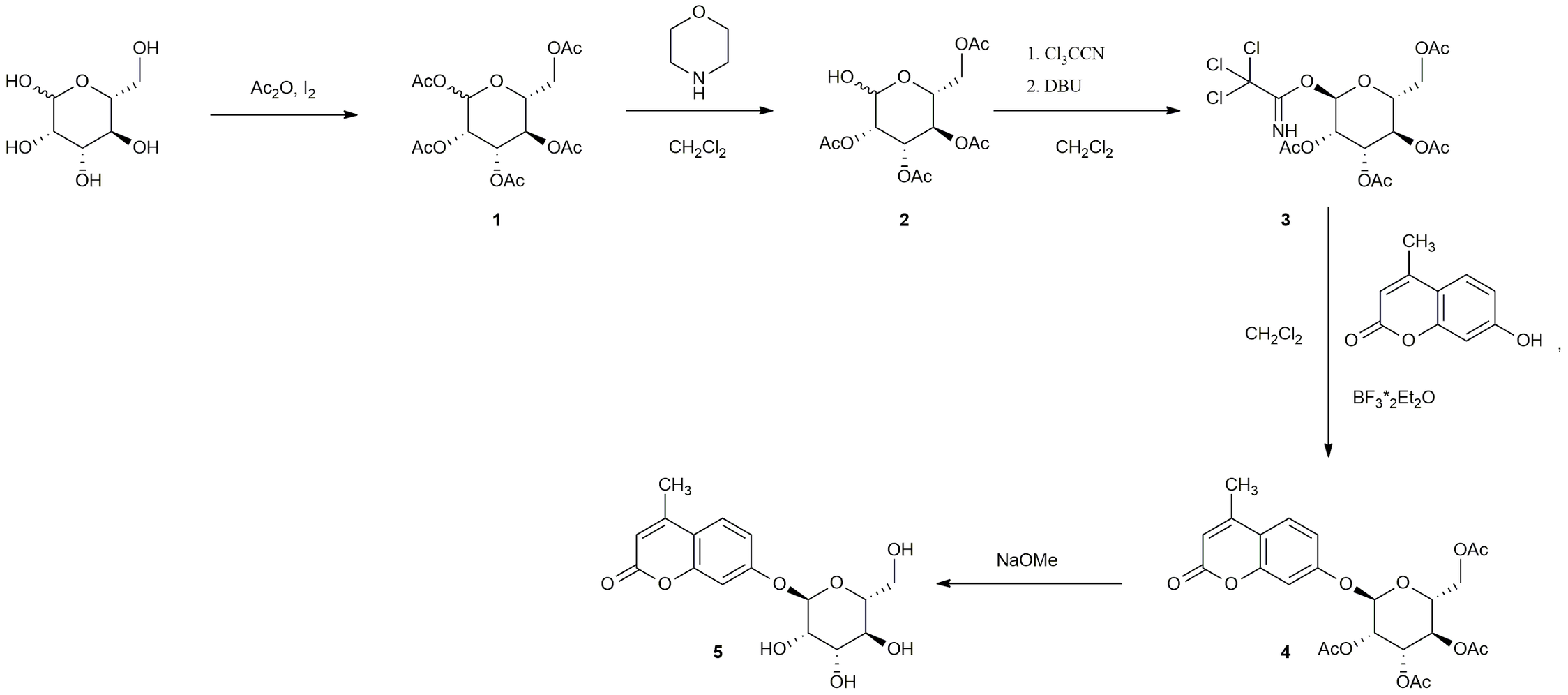

Supplement: S2 Fig — (TIF) [file pone.0216132.s004.tif]

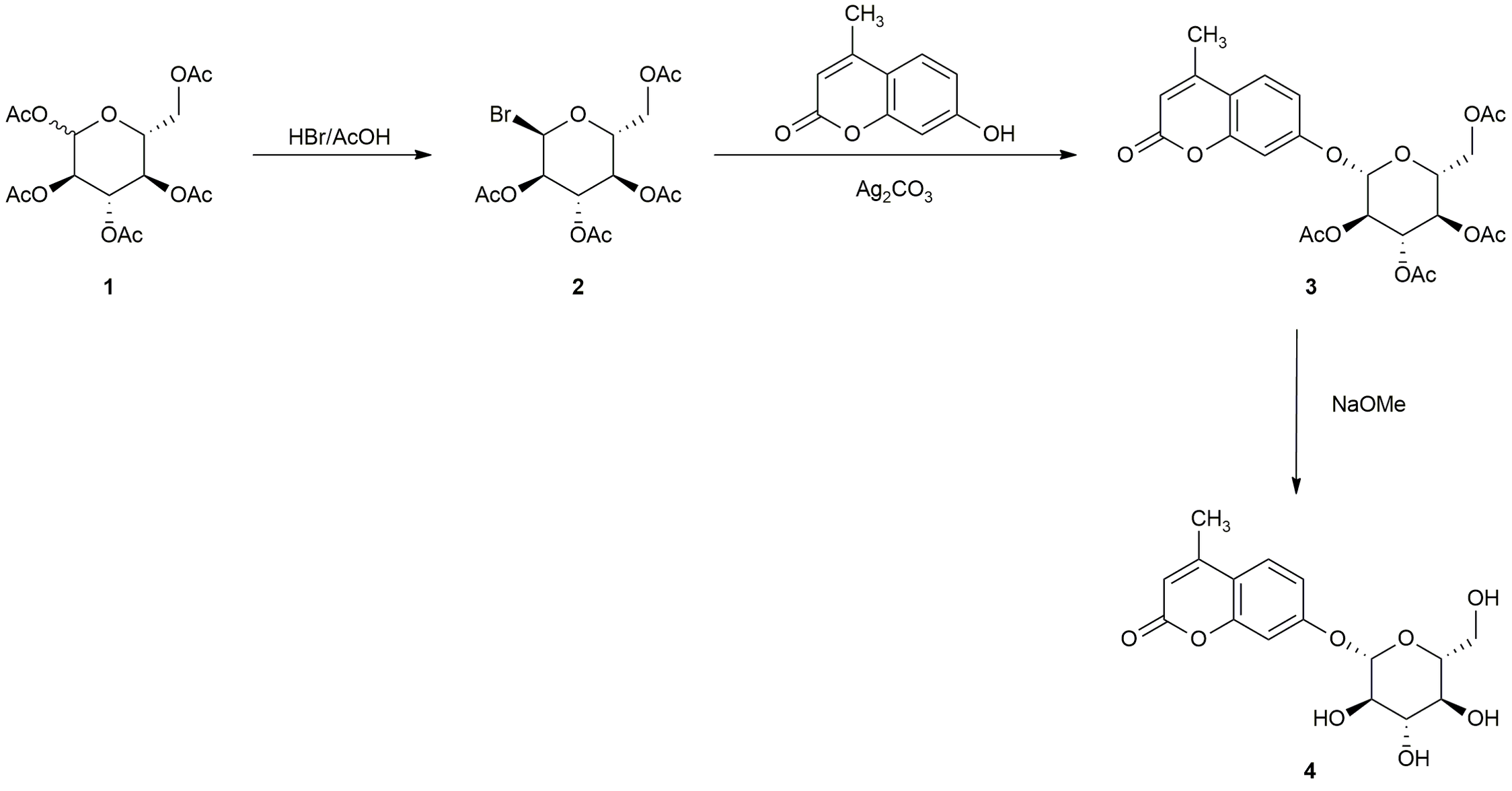

Supplement: S3 Fig — (TIF) [file pone.0216132.s005.tif]
